# Mapping Bornavirus encephalitis—A comparative study of viral spread and immune response in human and animal dead-end hosts

**DOI:** 10.1371/journal.ppat.1013400

**Published:** 2025-08-04

**Authors:** Yannik Vollmuth, Nicola Jungbäck, Przemyslaw Grochowski, Tatiana Mögele, Leonhard Stark, Niku S. Zarrabi, Jürgen Schlegel, Tina Schaller, Bruno Märkl, Kaspar Matiasek, Friederike Liesche-Starnecker

**Affiliations:** 1 Department of Neuropathology, Pathology, Medical Faculty, University of Augsburg, Augsburg, Germany; 2 Institute of Pathology, School of Medicine and Health, Technical University of Munich, Munich, Germany; 3 Department of Pediatrics, Dr. von Hauner Children’s Hospital, Ludwig-Maximilians- Universitaet Muenchen, Munich, Germany; 4 Pathology, Medical Faculty, University of Augsburg, Augsburg, Germany; 5 Department of Exercise Physiology, School of Medicine and Health, Technical University of Munich, Munich, Germany; 6 Section of Clinical and Comparative Neuropathology, Centre for Clinical Veterinary Medicine, Ludwig-Maximilians-Universitaet Muenchen, Munich, Germany; NIAID DIR: National Institute of Allergy and Infectious Diseases Division of Intramural Research, UNITED STATES OF AMERICA

## Abstract

Borna disease virus 1 (BoDV-1) has long been recognized as a cause of fatal encephalitis in animals and was only recently identified as a zoonotic pathogen causing a similar disease in humans. This study provides the first comprehensive comparative analysis of BoDV-1-induced neuropathology in human and animal end hosts, including horses, sheep, and alpacas. Using immunohistochemical analyses, we investigated the topographical distribution of BoDV-1 and inflammatory responses in the central nervous system across 19 cases. Key findings reveal distinct differences and overlaps between humans and animals. While humans exhibited heterogeneous patterns especially of the lymphocyte infiltration, animals displayed more species-specific inflammation and viral spread patterns. In horses, the hippocampus and basal ganglia were consistently affected, whereas sheep showed predominant involvement of the frontal cortex and stria olfactoria. Alpacas demonstrated a less uniform distribution but highlighted the brainstem and basal ganglia as critical sites. Intriguingly, across all species, a negative association was observed between lymphocyte infiltration and the number of BoDV-1-infected cells. These findings enhance our understanding of BoDV-1 pathogenesis and is a first step of cross-species comparison in unraveling disease mechanisms in BoDV-1 infection. Further research is warranted to elucidate the implications of these findings for therapeutic strategies and to explore the entry and dissemination routes of BoDV-1 in different hosts.

## Author summary

Borna disease virus 1 (BoDV-1) is a fatal virus that causes severe brain inflammation in both humans and animals. While the disease has been known in horses and sheep for centuries, it was only recently identified as a cause of deadly encephalitis in humans. In this study, we systematically compared BoDV-1 infections in humans, horses, sheep, and alpacas by analyzing autopsy/necropsy brain tissue. We found distinct patterns of viral spread and immune responses across species. Humans showed highly variable immune responses, while animals exhibited more species-specific patterns. Interestingly, across all species, higher viral protein values were associated with lower immune cell infiltration, suggesting that the immune response may help control viral spread. Our findings provide new insights into how BoDV-1 affects different hosts and may help to improve future diagnostic and treatment strategies. Understanding these species-specific differences could also shed light on how the virus enters and spreads in the brain.

## Introduction

Bornavirus encephalitis (BVE) is a rare but predominantly fatal disease affecting a wide range of mammals, including humans. Despite its long history in veterinary medicine, fundamental questions regarding its pathogenesis remain unanswered. Most critically, the immune-mediated inflammatory response appears to be the key driver of tissue damage, rather than direct viral cytotoxicity. Understanding the species-specific patterns of inflammation and viral distribution is essential to elucidate the pathomechanisms underlying the disease.

While “Borna disease” in animals – particularly in horses and sheep - has been recognized for over two centuries, its zoonotic potential was only confirmed in 2018 when Borna disease virus 1 (BoDV-1) was linked to severe, mostly fatal encephalitis in humans [1,2]. Retrospective studies have since identified BoDV-1 as the cause of previously unexplained human encephalitis cases [3]. Given the endemic distribution in parts of Germany and neighboring countries, BoDV-1 may account for a significant proportion of severe or fatal encephalitis cases of unknown origin in this area [4].

The bicolored white-toothed shrew (*Crocidura leucodon*) serves as the main natural reservoir of BoDV-1 [5–8]. In animal end hosts, transmission likely occurs through excretions from infected shrews with viral entry into the central nervous system (CNS) via the olfactory pathway [8–11]. A similar route is suspected in humans [12], yet the exact mechanism remains elusive. The observation that viral distribution varies between species [13], despite a presumed uniform olfactory entry, raises the question of whether additional, host-specific factors influence viral dissemination.

BoDV-1 itself is non-cytolytic, with tissue damage primarily driven by an immune response involving CD8 + T cells [14–17]. Due to the rarity of the disease and limited clinical awareness, the diagnosis is frequently delayed [18]. Currently, no approved treatments exist; only experimental studies have explored antiviral drugs *in vitro* and immunosuppressive therapy to mitigate immunopathology [3,14,19]. Early immunosuppression has been proposed as a potential strategy to influence disease progression in humans [20].

Despite the long history of Borna disease in animals and its recent identification in humans, no comprehensive comparative neuropathological studies have been conducted. Direct comparison of viral distribution and immune response across different species may provide crucial insights into host-specific pathogenesis, helping to distinguish generalizable patterns from species-dependent mechanisms. Furthermore, elucidating differences in inflammation and viral spread may clarify whether certain host factors influence disease severity or progression.

For this reason, this study systematically compares the topographical distribution of BoDV-1 and its associated inflammatory response in the CNS of human and animal dead-end hosts. Immunohistochemical and histomorphological analyses were performed on affected brains from humans and various mammals, including horses, sheep, and alpacas. Understanding species-specific differences and similarities may help uncover key mechanisms of disease transmission and pathogenesis, ultimately guiding future diagnostic and therapeutic approaches.

## Results

### BoDV-1 and inflammation distribution in human brain tissue

Details of the human cases, including distribution visualization and clinical course, have already been published by Vollmuth *et al.* [20]. Nevertheless, spatial distribution is provided in Table 1.

**Table 1:** Distribution of BoDV-1-positive cells and lymphocytes in human brains.

BoDV-1-positive cells								
	**P1**	**P2**	**P3**	**P4**	**P5**	**P6**	**P7**	**P8**
Frontal cortex	3.8	5.9	**7.1**	n.a.	0.0	12.1	0.2	0.0
Occipital cortex	5.9	1.7	0.0	13.5	3.6	12.4	0.2	0.0
Striatum	1.0	35.0	n.a.	11.7	7.6	9.8	5.2	1.2
Hippocampus	4.0	**38.0**	3.3	n.a.	n.a.	n.a.	0.3	0.5
Insular cortex	n.a.	n.a.	n.a.	n.a.	n.a.	n.a.	0.0	0.1
Mesencephalon	6.3	21.3	n.a.	5.9	n.a.	7.4	**16.4**	n.a.
Pons	n.a.	25.2	n.a.	n.a.	2.5	16.4	0.5	0.3
Medulla oblongata	**14.6**	23.8	n.a.	**21.1**	**8.2**	**27.9**	n.a.	**2.2**
Cerebellum	1.6	6.7	0.0	0.6	0.0	11.5	0.1	0.9
Stria olfactoria	n.a.	n.a.	n.a.	n.a.	n.a.	2.4	n.a.	0.1
Diffuse lymphocytes								
	**P1**	**P2**	**P3**	**P4**	**P5**	**P6**	**P7**	**P8**
Frontal cortex	**71.7**	1.1	1.0	n.a.	9.3	**44.4**	0.8	27.4
Occipital cortex	59.5	5.5	9.6	**28.9**	**40.0**	2.5	1.4	23.7
Striatum	61.3	1.4	n.a.	15.0	26.7	n.a.	12.5	14.8
Hippocampus	60.4	**6.6**	**16.2**	n.a.	n.a.	n.a.	5.1	7.3
Insular cortex	n.a.	n.a.	n.a.	n.a.	n.a.	n.a.	2.3	22.9
Mesencephalon	65.0	5.5	n.a.	2.2	n.a.	11.0	**26.9**	n.a.
Pons	n.a.	5.9	n.a.	n.a.	16.3	3.3	24.0	32.2
Medulla oblongata	16.7	3.1	n.a.	13.8	30.7	12.6	n.a.	**35.9**
Cerebellum	17.8	3.5	5.1	16.0	8.4	4.1	0.4	9.7
Stria olfactoria	n.a.	n.a.	n.a.	n.a.	n.a.	11.0	n.a.	1.9

n.a. = not analyzed. Values for BoDV-1-positive cells in %; values for lymphocytes in pASS. The highest value for each patient is marked in bold.

In short, the inflammation showed an interindividual heterogeneity in its regional distribution, with predominant involvement of the frontal cortex (patient (P) 1 and P6), hippocampus (P2 and P3), and brain stem, including the mesencephalon, pons, and medulla oblongata (P7 and P8). In two other cases, the inflammation was most prominent in the occipital cortex (P4 and P5).

Most BoDV-1-positive cells were detected in the brain stem, particularly in the medulla oblongata (P1, P4, P5, P6 and P8), and mesencephalon (P7), while in P2, viral distribution was highest in the hippocampus, followed by the basal ganglia, and brainstem. P3, with only four regions analyzed, is of limited interpretability.

Patients could be grouped into two categories based on the overall average percentage of BoDV-1-positive cells across all analyzed brain regions: P1, P3, P5, P7, and P8 displayed <5% infected cells, whereas P2, P4, and P6 showed significantly higher values (>10%). P2 had the highest proportion, with an average of 20% infected cells across all regions.

### Distribution pattern of BoDV-1-positive cells and lymphocytes in the CNS of horses, sheep, and alpacas

All animals exhibited marked inflammation, with CD3 + T-lymphocytes being more abundant than CD20 + B-lymphocytes.

Sheep had the highest total lymphocyte counts (mean: 43 lymphocytes per average of 10 screenshots (pASS) across all regions), followed by alpacas (mean: 25 pASS over all regions) and horses (mean: 20 pASS over all regions). In sheep, the highest lymphocyte counts were found in the stria olfactoria (61 lymphocytes pASS) and frontal cortex (56 lymphocytes pASS), whereas the cerebellum (24 lymphocytes pASS) and pons (26 lymphocytes pASS) were least affected. Alpacas exhibited the most severe inflammation in the brainstem, with alpaca (A) 1 showing 43 lymphocytes pASS in the pons, while A2 had 66 lymphocytes pASS in the medulla oblongata. Similar to sheep, alpacas showed only mild inflammation in the cerebellum (7 lymphocytes pASS). Horses displayed the highest lymphocyte counts in the hippocampus (45 lymphocytes pASS), followed by the basal ganglia (26 lymphocytes pASS), with the pons and insular cortex being the least affected (each 10 lymphocytes pASS).

**Fig 1:**
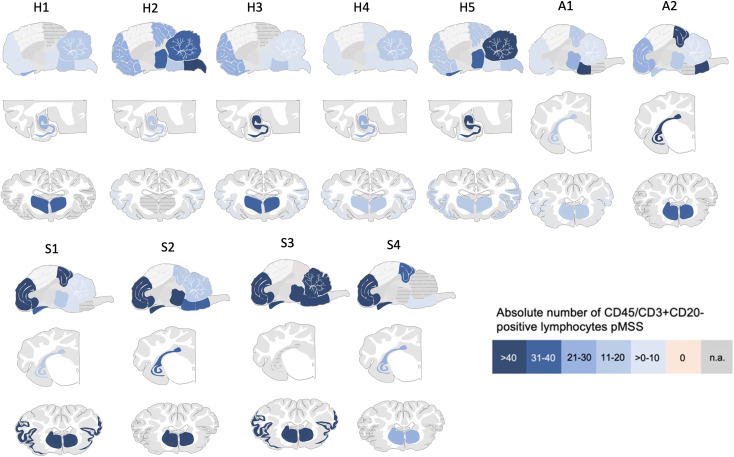
Lymphocyte distribution in horses, sheep and alpacas across brain regions. Species-specific patterns of inflammation are shown based on lymphocyte counts per brain region. Sheep exhibited strongest infiltration in the stria olfactoria and frontal cortex, alpacas in the brainstem, and horses in the hippocampus and striatum. H = horse, S = sheep, A = alpaca.

In summary, in horses, lymphocyte counts were highest in the hippocampus and basal ganglia; in sheep, elevated values were noted in the stria olfactoria, frontal cortex, and basal ganglia. Despite the limited sample size, both alpacas showed a shared pattern with prominent inflammation in the brainstem, basal ganglia, and hippocampus. A detailed spatial distribution for all included animals is provided in Fig 1 and Table 2.

**Table 2:** Distribution of lymphocytes in horses, sheep and alpacas.

	H1	H2	H3	H4	H5	S1	S2	S3	S4	A1	A2
Frontal cortex	3.5	29.2	22.1	2.3	17.7	**65.2**	54.4	57.3	**50.5**	7.8	29.3
Occipital cortex	n.a.	25.0	n.a.	2.4	11.5	41.6	14.7	52.2	34.9	15.4	46.2
Striatum	**40.7**	n.a.	33.3	11.8	18.4	42.2	**86.1**	63.1	28.6	20.1	37.0
Hippocampus	29.1	17.1	**57.6**	**30.5**	**90.8**	18.3	34.7	n.a.	26.9	13.9	55.3
Insular cortex	n.a.	8.2	8.3	8.0	17.4	41.4	n.a.	53.0	n.a.	9.7	n.a.
Mesen-cephalon	5.6	34.7	2.7	8.5	33.6	18.9	58.7	63.4	n.a.	21.4	17.2
Pons	12.6	12.6	15.4	1.1	10.5	9.9	34.3	54.8	6.0	**43.1**	n.a.
Medulla oblongata	5.4	**42.5**	5.9	10.4	19.5	n.a.	32.8	**105.7**	8.2	n.a.	**66.3**
Cerebellum	13.2	31.3	6.6	12.5	47.9	5.0	16.8	48.8	n.a.	9.3	5.6
Stria olfactoria	24.7	25.0	23.9	0.6	32.3	40.7	63.6	92.8	48.9	7.7	17.0

H = horse, S = sheep, A = alpaca, n.a. = not analyzed. Values for lymphocytes are given in pASS. The highest value for each animal is marked in bold.

### Distribution of BoDV-1-positive glial cells and neurons in the CNS of horses, sheep, and alpacas

**Fig 2:**
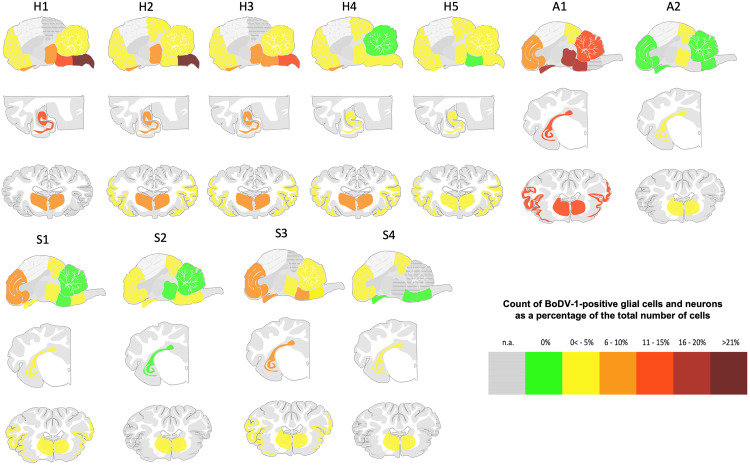
BoDV-1 distribution of horses, sheep and alpacas across brain regions. The most affected regions varied by species, with medulla oblongata and hippocampus frequently involved in horses, frontal cortex in sheep, and brainstem regions in alpacas. H = horse, S = sheep, A = alpaca.

Animals generally exhibited low proportions of BoDV-1-positive cells, except for A1. The highest percentage of infected cells was found in alpacas (mean: 7% positive cells cross all regions), followed by horses (mean: 5%) and sheep (mean: 2%; Fig 2 and Table 3).

**Table 3:** Distribution of BoDV-1positive cells in horses, sheep and alpacas.

Location	H1	H2	H3	H4	H5	S1	S2	S3	S4	A1	A2
Frontal Cortex	0.7	3.0	5.5	4.9	0.1	**10.3**	1.0	9.0	0.1	10.8	0.0
Occipital Cortex	n.a.	0.6	n.a.	1.0	0.1	3.8	1.0	n.a.	1.5	2.0	0.5
Striatum	9.5	4.4	7.7	**8.6**	0.2	0.9	0.6	1.7	0.1	11.8	0.1
Hippocampus	12.4	7.7	8.3	1.7	**2.8**	2.3	0.0	**9.4**	**3.5**	13.6	**2.6**
Insula Cortex	n.a.	4.3	3.8	0.7	0.8	1.2	n.a.	4.1	n.a.	14.1	n.a.
Mesence-phalon	7.5	6.3	10.8	4.1	0.1	0.3	0.0	0.9	n.a.	17.1	0.1
Pons	12.1	2.1	8.4	4.4	0.0	0.0	**2.3**	9.6	0.0	16.6	n.a.
Medulla oblongata	**35.6**	**11.7**	**12.7**	4.4	0.1	5.4	0.1	5.7	0.0	n.a.	0.0
Cerebellum	0.1	0.7	1.0	0.0	0.1	0.0	0.0	2.5	n.a.	18.2	0.0
Stria Olfactoria	7.3	8.8	7.4	6.5	2.0	3.2	1.7	6.1	0.0	**18.9**	0.0

H = horse, S = sheep, A = alpaca, n.a. = not analyzed. Values for BoDV-1-positive cells are given in %. The highest value for each patient is marked in bold.

In A1, the stria olfactoria was the most affected region (18% BoDV-1-positive cells), followed by the mesencephalon and pons (16%). The lowest infection rate was in the occipital cortex (2%). In A2, the hippocampus had a 2% infection rate, while the occipital cortex showed only minimal involvement (0.5%).

Among horses, the highest proportion of infected cells was in the medulla oblongata (12%), followed by the hippocampus (6%), stria olfactoria (6%), and striatum (5%). The cerebellum and occipital cortex were least affected (each <1%).

In sheep, the frontal cortex had the highest proportion of infected cells (4%), followed by the hippocampus and medulla oblongata (3%). The lowest levels of infection were observed in the striatum and cerebellum (each <1%).

### Clinical signs of the animals

Animals received symptomatic treatment after developing neurological signs (e.g., ataxia, circling, compulsive walking). A detailed overview of the clinical signs is provided in Table 4. These findings align with previously reported clinical presentations in BoDV-1-infected animals [1,21,22]. Of the 11 animals, 9 were euthanized due to severe clinical signs, one animal died during hospitalization, and the cause of death for another remained undocumented.

**Table 4:** Clinical signs of the animals.

Case-ID	Type of death	Clinical signs
H1	euthanasia	n.a.
H2	euthanasa	Fever, circle walking
H3	euthanasia	n.a.
H4	euthanasia	Ataxia hindquarters, behavioral disorder
H5	euthanasia	n.a.
S1	euthanasia	Pushing forward, somnolence, nystagmus
S2	n.a.	Pushing forward, running in circles, somnolent phases
S3	euthanasia	Emaciation, CNS disorders, ataxia
S4	euthanasia	Apathy, weak, separation from the flock, running in circles, chews empty in phases, ear defense delayed, eyelids pressed tightly together, lambs drink undisturbed from the mother
A1	euthanasia	Lack of defecation and urination, ileus, teeth grinding, inappetence, constant deterioration, lateral position, coma
A2	natural	Early in the morning gasping in supine position, exit in hospital

H = horse, S = sheep, A = alpaca.

### Comparison of the distribution pattern between humans and animals

A comparative analysis revealed that humans had the highest proportion of BoDV-1-infected cells (7.0% across all regions), followed by alpacas (6.8%), horses (5.4%), and sheep (2.4%). Interestingly, this pattern was reversed for lymphocyte infiltration: sheep exhibited the highest lymphocyte counts (42.5 lymphocytes pASS across all regions), followed by alpacas (25.3 lymphocytes pASS), horses (20.4 lymphocytes pASS), and humans (17.6 lymphocytes pASS).

Overall, high lymphocyte counts showed an association with low numbers of BoDV-1-infected cells, and vice versa. However, A1 presented an exception, exhibiting both high lymphocyte counts (16 lymphocytes pASS) and a high proportion of infected cells (13%). Despite this trend, no significant negative correlation between inflammation and viral distribution was observed when including all regions of all species (Spearman’s rho, p = 0.384).

**Fig 3:**
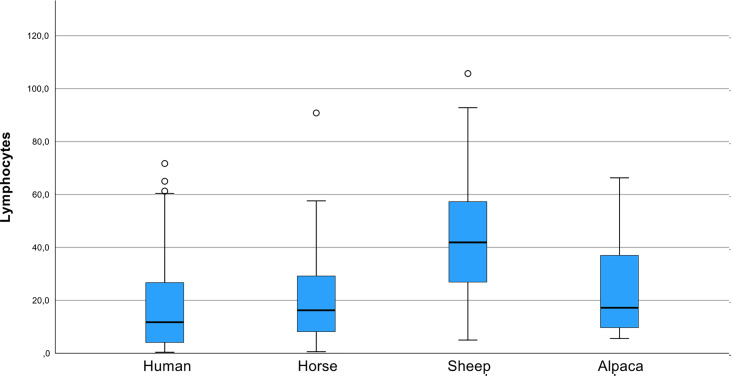
Boxplot comparison of lymphocyte counts pASS across species. Sheep showed the highest lymphocyte infiltration, whereas humans exhibited the lowest. Despite an inverse trend between lymphocyte counts and BoDV-1-infected cell proportions, no significant negative correlation was observed.

Sheep exhibited significantly higher lymphocyte counts than other species, particularly when compared to humans (p < 0.001) and horses (p < 0.001). Other interspecies differences were not statistically significant (Fig 3).

**Fig 4:**
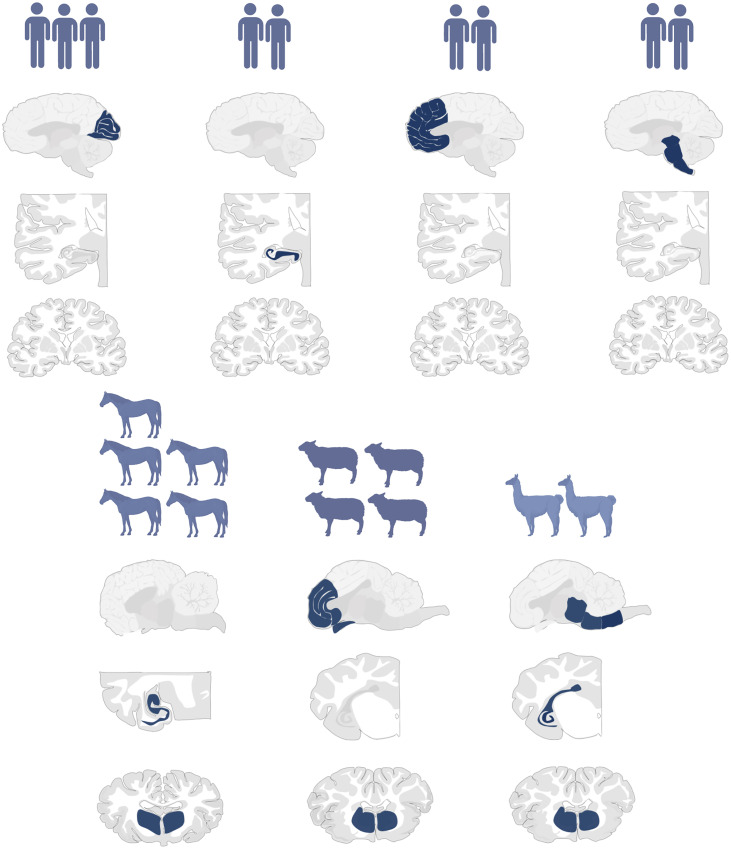
Overview of lymphocyte distribution in the CNS across species. Humans show heterogeneous patterns, while animals exhibit species-specific, consistent inflammation: hippocampus and striatum in horses, frontal cortex and stria olfactoria in sheep, and brainstem and hippocampus in alpacas. Created with Biorender.com.

Notably, humans showed a more heterogeneous inflammation pattern compared to animals. Horses exhibited a consistent inflammation pattern centered in the hippocampus, with some involvement of the basal ganglia. Sheep displayed dominant lymphocyte infiltration in the frontal cortex, striatum, and stria olfactoria (Fig 4). Alpaca cases, though limited, consistently showed inflammation in the brainstem, striatum, and hippocampus.

**Fig 5:**
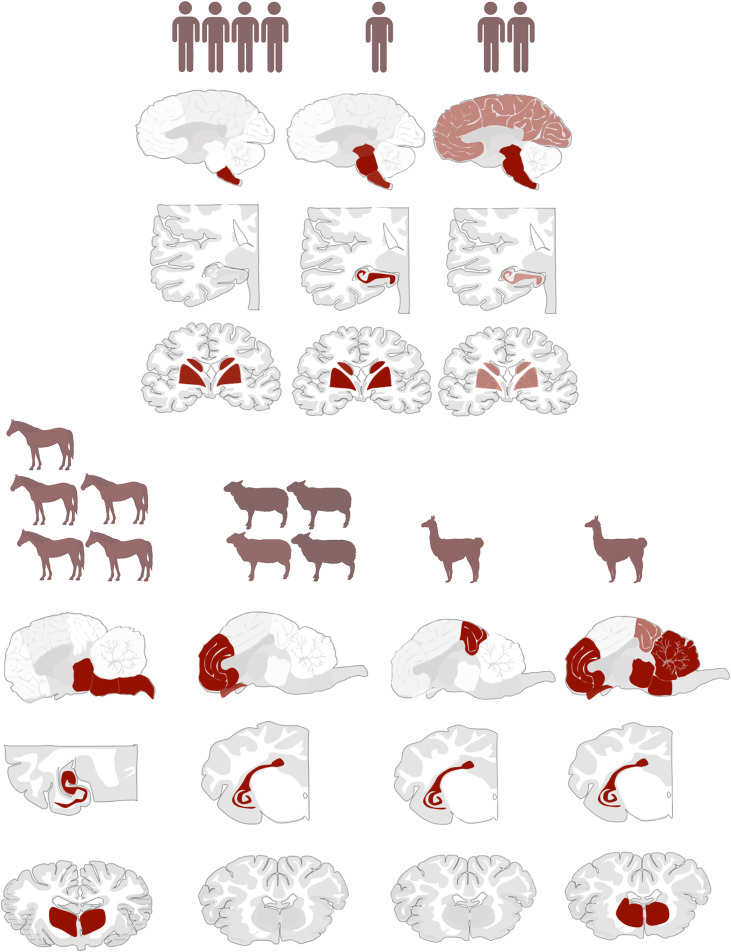
Overview of BoDV-1 virus distribution in the CNS across species. While humans and horses showed prominent involvement of the medulla oblongata and striatum, sheep were mainly affected in the frontal cortex. Alpaca data remain inconclusive due to limited sample size. Created with Biorender.com.

In human cases, BoDV-1-positive cells were concentrated in the medulla oblongata and basal ganglia (P4, P5, P7, P8), while other cases displayed a more diffuse distribution with primarily affected brainstem (P1, P6). P2 exhibited a spread pattern involving the hippocampus, basal ganglia, and brainstem, similar to that observed in horses. Horses (H1, H2, H3) had particularly high BoDV-1 concentrations in the medulla oblongata. In sheep, viral presence was most pronounced in the frontal cortex. Due to the small sample size, BoDV-1 distribution in alpacas remains inconclusive. A visual summary of virus distribution across species is provided in Fig 5.

## Discussion

Despite recent advances, BoDV-1 remains an elusive pathogen, with fundamental questions about its transmission routes, progression, and host-specific disease mechanisms still unresolved. While prior studies have provided insights into viral tropism and immune responses, a direct comparative analysis of BoDV-1 neuropathology across different host species has been missing. This study bridges that gap by offering the first in-depth comparison of BoDV-1 distribution and associated inflammation in human and animal dead-end hosts. By analyzing brain tissue from infected humans, horses, sheep, and alpacas, we identified species-specific patterns that could shed light on potential viral entry routes and immune responses.

In line with previous studies, strong BoDV-1 involvement in the hippocampus was found in four human cases, as well as in two sheep and one alpaca [23,24]. Similarly, the stria olfactoria showed prominent viral presence in three patients, one sheep and one alpaca supporting prior findings [23,25]. Notably, sheep exhibited a distinct distribution pattern, with limited BoDV-1 spread in the basal ganglia, and the primary focus on the frontal cortex and hippocampus. In horses, viral distribution was strongest in the hippocampus and medulla oblongata, consistent with the findings of Bilzer *et al.* [26]. The cerebellum remained the least affected area in all human and animal cases, confirming previous findings [24].

Conversely, the pronounced BoDV-1 involvement in the medulla oblongata, particularly in human and equine cases, raises important questions regarding the potential routes of viral entry. Despite an assumed shared olfactory entry [27], the distinct regional involvement observed – particularly the pronounced medullary involvement in humans and horses versus the more cortical pattern in sheep – raises the possibility of species-specific neuroanatomical factors influencing viral dissemination. Alternative entry routes, such as peripheral nerve invasion or hematogenous spread, cannot be ruled out. This discrepancy underscores the complexity of BoDV-1 pathogenesis and highlights the need for additional experimental models to validate the relevance of different transmission pathways.

Lymphocyte distribution also varied considerably across species. While human cases showed diverse inflammatory patterns, horses demonstrated a more uniform focus on the hippocampus and basal ganglia, and sheep exhibited strong lymphocytic infiltration in the stria olfactoria, frontal cortex, and basal ganglia. In alpacas, the available data were limited, but both cases showed increased lymphocyte counts in the brainstem, basal ganglia, and hippocampus.

Treatment effects likely contributed to the neuropathological patterns observed in human cases. As recently reported in a detailed clinical study of the same cohort [28], all but one patient received immunosuppressive therapy – with variable timing and duration – which is likely to have modulated the extent and composition of CNS inflammation, and possibly contributing to the relatively low lymphocyte counts observed in some patients despite high viral loads. These confounding effects – in addition to differences in disease duration and supportive measures – must be considered when comparing viral and inflammatory patterns across species.

For the animal cases, treatment histories could unfortunately not be reconstructed due to missing clinical records. However, it can be assumed that, if treatment was given at all, it was limited to symptomatic care. The absence of immunosuppressive interventions may explain the more uniform patterns of viral distribution and inflammation observed within each animal species.

This more homogenous distribution in animals contrasts with the considerable interindividual variability observed in the human cohort, with some cases exhibiting a strong BoDV-1 presence in the basal ganglia and medulla oblongata, while others displayed a more diffuse distribution or prominent involvement of the hippocampus. The equine cohort showed a more consistent pattern, with predominant viral spread in the hippocampus, basal ganglia, and brainstem. In contrast, the sheep specimens exhibited a unique viral distribution, primarily affecting the frontal cortex and hippocampus while largely sparing the basal ganglia.

Given the variability in treatment status across cases, any direct comparison between treated and untreated individuals must be interpreted with caution. Immunosuppressive therapies administered in most human cases may have significantly altered both viral load and inflammatory responses, whereas animal cases likely reflect the natural course of infection, as no specific antiviral or immunosuppressive treatments were documented. These differences introduce an important limitation to the cross-species comparison and highlight the need for controlled experimental studies to systematically assess treatment effects on BoDV-1 pathogenesis.

Nevertheless, the variability in BoDV-1 distribution across species raises the question if the observed patterns translate into clinical signs and symptoms. In particular, the pronounced viral involvement of the medulla oblongata and basal ganglia in humans and horses may explain observed neurological deficits, as these structures are critical for motor control and autonomic functions. In sheep, where viral spread is concentrated in the frontal cortex and hippocampus, alterations in behavior or executive function might predominate. However, a direct correlation between neuropathological findings and clinical presentation remains challenging in this study. The limited case numbers reduce statistical power, and in human cases, historical medical records were often incomplete, particularly for older cases, complicating clinicopathological interpretation.

For the human cases included in this study, the clinical course has been reported previously [28], and was characterized by a prodromal phase with non-specific flu-like symptoms (e.g., fever, fatigue, dizziness), followed by rapid neurological deterioration. Most patients developed disorientation, ataxia, seizures, and eventually a deep coma with brainstem failure, leading to death on average 30 days after symptom onset. This pattern broadly corresponds to the postmortem distribution of BoDV-1 in the hippocampus and brainstem.

While a comparative interpretation of neuropathological findings and clinical signs across species would be highly valuable, such an analysis is limited by the fragmentary clinical documentation available for several human cases and the inherent challenges of systematically recording neurological signs in animals. These constraints restrict the possibility of a meaningful cross-species correlation and should be considered when interpreting the observed patterns.

Future studies should aim for a prospective design with standardized neurological evaluations to better establish these relationships.

Overall, humans exhibited higher viral loads but lower lymphocyte counts compared to animals. This discrepancy might reflect to prolonged disease courses in human patients, facilitated by intensive care and immunosuppressive treatments, allowing continued viral replication despite immune system activation. In contrast, animals often exhibited acute, severe disease courses with strong immune responses, potentially leading to faster viral clearance.

In four cases, the human specimens showed a focus of the virus in the basal ganglia and the medulla oblongata. In two cases, a diffuse distribution was found and in one case, the hippocampus as well as the basal ganglia and the brain stem were heavily involved. In the horses, the virus spread was also focused on the hippocampus, the basal ganglia and the brain stem. The comparison of the findings indicates that the virus distribution in equine specimens was similar to that in human specimens. Differences were found in the sheep brain preparations. These showed a focus of the virus in the frontal cortex and the hippocampus with a simultaneous omission of the basal ganglia. The reason for this could be an alternative portal of entry. The lymphocyte distribution in the human cases was not uniform. In three cases the inflammation focused on the occipital cortex, in two cases on the frontal cortex. The brain stem and the hippocampus were involved in two further cases each. Differences in lymphocyte distribution may be influenced by treatment history, such as the use of immunosuppressive therapies, though specific data are limited. Furthermore, different portals of entry could also be conceivable.

A striking observation was that, in several cases, regions with high viral presence showed a reduced lymphocytic count. One possible explanation for this is that as the disease progresses, initially affected regions become heavily infiltrated with lymphocytes, leading to an immune-mediated clearance of BoDV-1-infected cells. The virus may thus be forced to spread to other, previously unaffected regions. This hypothesis aligns with our findings, as in some human cases, BoDV-1-positive cells were predominantly detected in the medulla oblongata and basal ganglia, while the primary inflammatory response was located elsewhere. This observation raises the possibility that viral distribution patterns may vary over the course of infection, potentially as a result of immune-driven displacement of the virus. This phenomenon may reflect a time-dependent redistribution of the infection, where immune mechanisms gradually eliminate the virus from the initially affected regions, allowing it to persist in immune-privileged or less responsive areas of the CNS. If so, this could have implications for therapeutic timing, suggesting that early intervention might be crucial in preventing extensive viral dissemination.

In future studies, combining histomorphological data with spatial transcriptomics or deep viral sequencing could yield more detailed insights into local host–virus interactions and tissue-specific immune responses. Moreover, a serial sacrifice experiment in animal models might clarify the temporal sequence of viral dissemination and immune activation. Such longitudinal studies would be especially valuable to validate the hypothesized dynamics of immune-mediated viral clearance and spatial redistribution during disease progression.

While our comparative analysis reveals species-specific patterns of BoDV-1 distribution and inflammation, several important limitations inherent to the retrospective study design must be considered. Case numbers per species were small, limiting statistical power and precluding robust interspecies comparisons. Furthermore, individual differences in the disease course, such as time point of infection, disease duration, viral exposure dose, and clinical interventions (e.g., corticosteroid or other immunosuppressive therapies in human cases), are not uniformly documented. These factors may have significantly influenced both viral distribution and immune response. In addition, species-specific anatomical and immunological characteristics could also confound the observed patterns. As such, the results presented here should be interpreted as descriptive and hypothesis-generating. Prospective, controlled studies are needed to disentangle the complex interactions between viral pathogenesis, host anatomy, immune response, and therapeutic interventions.

Despite these limitations, this study provides the first detailed comparison of BoDV-1 encephalitis in humans and animals, revealing species-specific patterns of viral spread and immune response with clear results regarding the distribution patterns within different dead-end hosts. Our findings highlight the need for further research on BoDV-1 entry routes and immune interactions to better understand disease progression.

## Materials and methods

### Ethic statement

The study was approved by the local ethics committees of the Technical University of Munich (approval number 577/19 S) and the Ludwig-Maximilians-Universitaet Munich, which serves as the responsible ethics committee for the Augsburg University Hospital (approval number 23-0267). Formal consent was not obtained as the study is a retrospective analysis of autopsy material from deceased individuals. In all cases, informed consent for autopsy had been provided by the legal next of kin.

### Material

The cohort includes eight deceased human patients who all died of BVE between 2013 and 2022, as well as 11 animal cases (five horses, four sheep, and two alpacas) documented between 1995 and 2018. The average age at disease in humans was 43 years (range: 12–74 years; median: 43 years). In animals, the average age of disease onset was six years (range: 1–17 years; median: 4 years). Species-specific data are provided in Table 5, and detailed information on all 19 individuals is available in Table 6.

**Table 5:** Demographic data on all species.

Species	Number of cases	Gender m/w	Age (years)			Duration of illness (days)		
			Mean	Median	Range	Mean	Median	Range
human	8 (7^*^)	2/5	43	43	12-74	31	39	23-94
horse	5	1/4	8.6	9.0	1-17	n.a.	n.a.	n.a.
sheep	4	2/2	2.5	2.0	2-4	n.a.	n.a.	n.a.
alpaca	2	1/1	5.5	5.5	4-7	n.a.	n.a.	n.a.

n.a. = not available.

* For human cases, the number is indicated as 8 (7), reflecting that while 8 individuals were included, demographic information was only available for 7 patients.

**Table 6:** Demographic data on included humans and animals.

Case-ID	Species	Age (years)	Gender	Month of illness	Duration of illness (days)
P1	human	74	w	January	94
P2	human	21	w	February	40
P3	human	43	w	January	26
P4	human	70	m	November	23
P5	human	12	m	August	31
P6	human	n.a.	n.a.	n.a.	n.a.
P7	human	13	w	November	33
P8	human	71	w	July	29
H1	horse	1	w	June	n.a.
H2	horse	9	w	March	n.a.
H3	horse	6	w	March	n.a.
H4	horse	10	w	April	n.a.
H5	horse	17	m	September	n.a.
S1	sheep	4	m	January	n.a.
S2	sheep	2	m	March	n.a.
S3	sheep	2	w	July	n.a.
S4	sheep	2	w	June	n.a.
A1	alpaca	4	m	April	n.a.
A2	alpaca	7	w	June	n.a.

n.a. = not available.

All human cases except for P6 were published in detail by Vollmuth *et al.* [20]. Cases P1, P2, and P7 were additionally described by Liesche *et al.* and Finck *et al.* [13,29].

Animal cases were selected based on the availability of sufficient brain tissue, with focus on the ten anatomical regions analyzed in this study (see below). These cases therefore represent those with the most comprehensive tissue sampling across key brain areas. Although interspecies differences of brain anatomy are recognized, the selection of functionally analogous regions was intended to maximize comparability between species.

A total of 19 brains from BoDV-1-infected human and animal hosts were examined. The brain autopsies/necropsies had already been carried out according to the standard protocol for the respective species before this study. In brief, the brains were dissected after formalin fixation. Samples were preserved in formalin-fixed, paraffin-embedded blocks. While minor variations in initial tissue processing cannot be entirely ruled out due to the multicentric origin of the cohort, all samples were handled according to widely established standards, and any differences are considered negligible.

To ensure comparability in this study, ten brain regions were selected based on availability across all cases: frontal lobe, occipital lobe, striatum, hippocampus, insula, mesencephalon, pons, medulla oblongata, cerebellum, and stria olfactoria. The availability of these regions for each individual is summarized in Table 7.

**Table 7:** Examined and available brain regions.

Case-ID	FL	OL	Str	Hc	IL	Me	Po	MO	Cb	SO	Number
P1	X	X	X	X		X		X	X		7/10
P2	X	X	X	X		X	X	X	X		8/10
P3	X	X		X					X		3/10
P4		X	X	X		X	X	X	X		7/10
P5	X	X	X				X	X	X		6/10
P6	X	X	X			X	X	X	X	X	8/10
P7	X	X	X	X	X		X		X		7/10
P8	X	X	X	X	X		X	X	X	X	9/10
H1	X		X	X		X	X	X	X	X	8/10
H2	X	X	X	X	X	X	X	X	X	X	10/10
H3	X		X	X	X	X	X	X	X	X	9/10
H4	X	X	X	X	X	X	X	X	X	X	10/10
H5	X	X	X	X	X	X	X	X	X	X	10/10
S1	X	X	X	X	X	X	X	X	X	X	10/10
S2	X	X	X	X		X	X	X	X	X	9/10
S3	X	X	X	X	X	X	X	X	X	X	10/10
S4	X	X	X	X			X	X		X	7/10
A1	X	X	X	X	X	X	X		X	X	9/10
A2	X	X	X	X		X		X	X	X	8/10

FL = frontal lobe, OL = occipital lobe, Str = Striatum, Hc = Hippocampus, IL = insula, Me = mesencephalon, Po = pons, MO = medulla oblongata, Cb = cerebellum, SO = stria olfactoria.

### Immunohistochemistry

For immunohistochemistry, tissue sections were cut into 2-µm-thick slides and dried for 30 minutes. Epitope retrieval was performed according to antibody-specific protocols. For CD3 staining, retrieval was carried out in TRIS/EDTA buffer (pH 9.0) in a microwave at 700 watts for 2x 10 min. No pretreatment was required for CD20. For BoDV-1 and CD45 staining, citrate buffer (pH 6.0) was used in a steamer for 20 min.

Following pretreatment, tissue sections were incubated with the primary antibodies. The following antibodies were used: anti-CD3: polyclonal, rabbit, 1:100 dilution (Dako, Denmark), incubated for 60 min at room temperature; anti-CD20: polyclonal, rabbit, 1:400 dilution (Thermo Fisher, USA), incubated for 60 min at room temperature; anti-CD45: monoclonal, mouse, 1:120 dilution (clone 2B11 & PD7/26; DCS, Germany), incubated overnight at 4°C; anti-BoDV-1 nucleoprotein (antibody Bo18): monoclonal, mouse, 1:1000 dilution, as previously described [13,30–32].

A biotinylated secondary antibody diluted in blocking buffer was applied at room temperature for 50 min for Bo18 and CD45, and for 30 min for CD3 and CD20, followed by incubation with ABC reagent (Vector Laboratories, USA). The antibody binding was visualized using diaminobenzidine-hydrochloride (DAB) reagent (Vector Laboratories, USA). Counterstaining was performed with haematoxylin. Positive controls were included in each run for quality assurance.

### Image analysis

The BoDV-1- and CD45/CD3/CD20-stained slides were digitized using the Leica Aperio AT2 scanner at up to x200 magnification, with images uploaded to the Aperio eSlides Manager. For each slide, 10 screenshots (924 × 638 pixels, equivalent to 0.6 mm²) after a standardized schema (2x two fields at the top and three at the bottom) were captured. CD45/CD3/CD20-stained images were analyzed using Image J, adjusting brightness, saturation, hue, cell size, and circularity to optimize detection of CD45 + /CD3 + /CD20 + inflammatory cells. For precise adjustment parameters see S1 Table. Lymphocyte counts were averaged across the 10 screenshots (referred to as pASS: per average of 10 screenshots). Each case underwent quality control, with manual recounting every third analyzed region.

**Fig 6:**
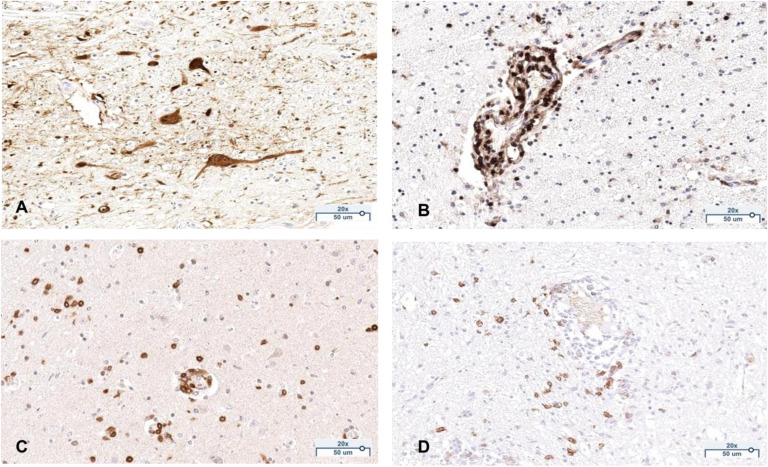
Exemplary images of the immunohistochemical staining. BoDV-1 **(A)**, CD45 **(B)**, CD3 **(C)**, CD20 **(D)**. All images are taken at a 200x magnification. All scale bars: 50 µm.

Automated analysis of BoDV-1 immunohistochemistry was not feasible due to color contrast variations, likely caused by differing infection levels. Therefore, positive and negative glial cells and neurons were manually counted, with averages calculated across 10 screenshots. Fig 6 provides representative examples of all immunohistochemical stainings.

### Statistical analysis

Statistical analysis was performed using IBM SPSS Statistics Version 27.0. Since the patient cohort data were not normally distributed, non-parametric tests were applied. The Mann-Whitney U test was used to analyze two variables. For the comparison of more than two variables, the Kruskal-Wallis H test was performed. Correlation analyses were performed using the Spearman-Rho test. A significance level of p ≤ 0.05 was applied.

### Use of artificial intelligence (AI) tools

During the manuscript preparation, OpenAI’s ChatGPT (GPT-4) was used to assist with the writing process. Specifically, selected passages within the Introduction, Discussion, and Methods sections were drafted or revised with the support of this Large Language Model to improve structure, clarity, and linguistic precision. The tool was not used to generate scientific ideas, interpret results, or formulate conclusions. All scientific content, and interpretations represent the authors’ own intellectual contributions. The authors critically reviewed and validated all AI-assisted suggestions.

## Supporting information

S1 Table Image J settings for the semi-automated analysis of CD45, CD3 and CD20. (DOCX)
