# Supplementary material for: Mapping Bornavirus encephalitis—A comparative study of viral spread and immune response in human and animal dead-end hosts
Source: PLoS Pathog. 2025 Aug 4;21(8):e1013400. doi: 10.1371/journal.ppat.1013400 (PMC12338802; doi:10.1371/journal.ppat.1013400)
Supplement: S1 Table — (DOCX) [file ppat.1013400.s001.docx]

**Supporting information**

**S1 Table**

| Image J  Settings | Hue | Saturation | | Brightness | Size | Circularity |
| --- | --- | --- | --- | --- | --- | --- |
| CD45 | min=0  max=80 | min=0  max=255 | min=0  max=50 | | 35-  Infinity | 0,2-1 |
| CD3 | min=0  max=160 | min=80  max=255 | min=0  max=255 | | 70-  450 | 0,2-1 |
| CD20 | min=0  max=155 | min=70  max=255 | min=0  max=255 | | 35-  Infinity | 0,1-1 |
